# Patients Satisfaction with Dental Care in the Slovak Republic: A Cross-sectional Questionnaire Study

**Published:** 2017-08

**Authors:** Martin SAMOHÝL, Anna NÁDAŽDYOVÁ, Katarína HIROŠOVÁ, Diana VONDROVÁ, Daniela KRAJČOVÁ, Jana JURKOVIČOVÁ

**Affiliations:** 1. Institute of Hygiene, Faculty of Medicine, Comenius University, Bratislava, Slovak Republic; 2. Dept. of Stomatology and Maxilofacial Surgery, Faculty of Medicine, Comenius University, Bratislava, Slovak Republic

## Dear Editor-in-Chief

The health care quality (HCQ) is a multidimensional concept (1) that includes the degree to which health services for individuals and populations increase the likelihood of desired health outcomes and are consistent with current instruments knowledge. Many health care organizations (departments) are criticized due to low patient satisfaction, and this highlights that holistic patient care is essential (2). A network of dental clinics falling within a system of public and private health care facilities is arranged in such a way, to that its composition and distribution creates sufficient conditions for providing state-guaranteed health care.

The aim of the study was to determine the level of HCQ satisfaction in dental clinic patients in terms of general satisfaction, technical quality, communication, interpersonal and financial aspects, time spent with the doctor, access/availability/convenience, and overall satisfaction from the patient viewpoint.

In this cross-sectional study, the standardized “Patient Satisfaction Questionnaire” PSQ III (long-form) was used (3). Respondents were free to choose an answer on a scale from 1 to 5 – to what extent they agree with each statement 1) strongly agree, 2) agree, 3) not sure, 4) disagree, 5) strongly disagree. The statements in the standardized questionnaire are divided into seven subscales: general satisfaction (6 questions), technical quality (10 questions), interpersonal aspects (7 questions), communication (5 questions), financial aspects (8 questions), time spent with the doctor (2 questions), and access/availability/convenience (12 questions); we evaluated the overall satisfaction (50 questions altogether) as well. Answers to some questions where strong agreement means the maximum satisfaction with the HCQ had to be rescaled (strongly agree: 5, agree: 4, disagree: 2, strongly disagree: 1) to obtain a unified HCQ score: 1= maximum dissatisfaction with the HCQ, 5= maximum satisfaction with the HCQ. The highest average value means the highest level of HCQ satisfaction. The study sample was recruited from patients attending dental surgeries in the capital of Slovakia and in several small towns in western and eastern regions of Slovakia; questionnaires were collected from Nov 2014 to Apr 2015. In total, 433 completed questionnaires were collected. The questionnaire was anonymous and a privacy policy was respected, participation in the study was voluntary. The data was analyzed using the statistical program SPSS (Chicago, IL, USA).

The highest rates of patients HCQ satisfaction (expressed as a mean score and as a percentage of maximum possible score) were achieved in the subscale time spent with doctor (2.81±0.65; 56.2%) and financial aspect (2.80±0.26; 55.9%); the lowest rate was found in the subscale general satisfaction (2.54±0.35; 50.8%). The overall satisfaction rate (i.e. an average value of all subscales) was only 2.69±0.20 or 53.8% (Table 1).

**Table 1:** Actual score, level of satisfaction (% of maximum possible score) and mean score in each subscale of HCQ satisfaction, and in overall satisfaction (n=433)

**PSQ-50 scale**	**No. items**	**Maximum possible score**	**Actual score**	**Level of satisfaction (%)**	**Mean score x (SD)**
General Satisfaction	6	30	15.3	50.8	2.54 (0.35)
Technical Quality	10	50	28.9	51.7	2.59 (0.26)
Interpersonal Aspect	7	35	19.1	54.5	2.73 (0.40)
Communication	5	25	13.2	52.7	2.64 (0.32)
Financial Aspect	8	40	22.1	55.9	2.80 (0.26)
Time Spent with Doctor	2	10	5.6	56.2	2.81 (0.65)
Access/Availability/Convenience	12	65	35.7	54.9	2.75 (0.27)
Overall Satisfaction	50	255	138.6	53.8	2.69 (0.20)

Patient satisfaction found in the subscale time spent with doctor was lower than other studies (4), however, in our sample patients declared the highest level of satisfaction right in this subscale. The study sample was recruited from patients attending dental surgeries where health care is partly covered by health insurance companies and the majority of the patients came from Bratislava (the capital of Slovakia) region with the highest average income. Some answers in this subscale could be skewed because the kind of medical examination was not taken into account. HCQ in this subscale was assessed as poor when compared (5). The mean overall satisfaction score in our study was lower when compared with study, that reported in ophthalmological patients the mean overall score 4.0 (4). This can be explained by differences in demographical, behavioral, psychological and cultural factors.

A possible limitation of this study is the sample size and its representativeness, which could pose problems in terms of generalizing the results. However, it could help to identify those aspects requiring improvement and establishing which improvements are needed. It is also important to identify the reasons for dissatisfaction and to complement this information.
